# Circadian Clock Control of Nox4 and Reactive Oxygen Species in the Vasculature

**DOI:** 10.1371/journal.pone.0078626

**Published:** 2013-10-25

**Authors:** Ciprian B. Anea, Maoxiang Zhang, Feng Chen, M. Irfan Ali, C. Michael M. Hart, David W. Stepp, Yevgeniy O. Kovalenkov, Ana-Maria Merloiu, Paramita Pati, David Fulton, R. Daniel Rudic

**Affiliations:** 1 Department of Pharmacology & Toxicology, Georgia Regents University, Augusta, Georgia, United States of America; 2 Vascular Biology Center, Georgia Regents University, Augusta, Georgia, United States of America; 3 Department of Physiology, Georgia Regents University, Augusta, Georgia, United States of America; 4 Department of Medicine, Atlanta Veterans Affairs and Emory University Medical Center, Atlanta, Georgia, United States of America; University of Alabama at Birmingham, United States of America

## Abstract

Recent studies have shown that circadian clock disruption is associated with pathological remodeling in the arterial structure and vascular stiffness. Moreover, chronic circadian disruption is associated with dysfunction in endothelial responses and signaling. Reactive oxygen species have emerged as key regulators in vascular pathology. Previously, we have demonstrated that circadian clock dysfunction exacerbates superoxide production through eNOS uncoupling. To date, the impact of circadian clock mutation on vascular NADPH oxidase expression and function is not known. The goal in the current study was to determine if the circadian clock controls vascular Nox4 expression and hydrogen peroxide formation in arteries, particularly in endothelial and vascular smooth muscle cells. In aorta, there was an increase in hydrogen peroxide and Nox4 expression in mice with a dysfunctional circadian rhythm (Bmal1-KO mice). In addition, the Nox4 gene promoter is activated by the core circadian transcription factors. Lastly, in synchronized cultured human endothelial cells, Nox4 gene expression exhibited rhythmic oscillations. These data reveal that the circadian clock plays an important role in the control of Nox4 and disruption of the clock leads to subsequent production of reaction oxygen species.

## Introduction

The circadian clock is a molecular mechanism that confers 24-hour variations in gene expression and function, allowing a better correlation between daily changes in the body and the environment. Although recent studies unravelled the negative consequence a broken clock can have on cardiovascular physiology, endothelial function, and vascular disease [1-13], the mechanisms by which the circadian clock influences the onset and progression of vascular dysfunction remain unclear. Mice with mutation of circadian clock components, such as the transcription factor Bmal1, exhibit acute vascular dysfunction with aberrant chronic vascular responses in remodelling [2]. Vascular stiffness and increased in matrix metalloproteinase (MMP) activity which is observed in circadian clock knockout mice [1] has been correlated with increased levels of reactive oxygen species [14]. Recently, we have found superoxide levels are increased in vessels from Bmal1-KO mice, due to uncoupling of eNOS[15]. Another significant source of reactive oxygen species are NADPH oxidases, of which the Nox4 isoform is unique in its ability to form hydrogen peroxide [16,17]. Herein, we demonstrate that Nox4 expression and function in arteries from Bmal1-KO mice is increased with Nox4 gene exhibiting oscillatory circadian expression in human endothelial cells. Moreover, Nox4 promoter was directly activated by circadian transcription factors Bmal1 + NPAS or Bmal1 + Clock. In conclusion this study describes a novel role for the circadian clock in modulation of vascular ROS by activation of Nox4 promoter.

## Materials and Methods

### Mice

All experiments were conducted in accordance with the National Institutes of Health Guide for the Care and Use of Laboratory Animals and approved and monitored by the Georgia Regents University, Institutional Animal Care and Use Committee. Wild-type (WT) litter-mate control and congenic Bmal1-KO mice (22-32 g, Jackson Labs, ages 6-16 weeks) were used in all studies. Mice were housed under standard 12 hour light/dark conditions (LD). 

### Western Blotting

Aortas were dissected, pulverized under liquid nitrogen, and protein was extracted. Protein concentration was determined by BCA kit and loaded on SDS-PAGE gels. Nox4 protein expression was detected with rabbit anti-mouse polyclonal antibodies (Abcam, cat no. ab60940), followed by enhanced chemiluminescence (ECL kit, Amersham). Densitometry was performed using Image J software.

### Real time quantitative PCR

Total RNA vas isolated from either aortae or human aorta endothelial cells cultured in vivo using RNA-easy extraction kit. cDNA was synthesized using iScript cDNA synthesis kit (Bio-Rad, Hercules, CA). Quantitative real-time PCR was carried out using a SYBR Green Supermix (Bio-Rad, Hercules, CA) and primers to Nox4 (forward primer, TGTTGCATGTTTCAGGTGGT; reverse, AAAACCCTCGAGGCAAAGAT) and Nox1 (forward primer, CATGGCCTGGGTGGGATTGT; reverse, TGGGAGCGATAAAAGCGAAGGA). Results were quantified by ΔΔCT method and results normalized with respect to GAPDH or 18S rRNA.

### Promoter Activity Assay

COS-7 cells were transfected and the total amount of expression plasmid transfected per well was kept constant by adding varying amounts of empty vector. The human Nox4 promoter cloned in PGL4.10-basic luciferase reporter vector (Promega, Madison, WI) (nucleotides −718 to +3 of the Nox4 gene) as described [18] was transfected into COS cells with control, Bmal1, Npas2, or Clock plasmids. At 24-h post-transfection, promoter activity was measured by a dual luciferase system using firefly luciferase normalized to Gaussian luciferase (Promega, New England Biolabs).

### Endothelial and Smooth Muscle Cell Isolation

Aortae isolated from 6-8 week old mice were used to isolate endothelial and smooth muscle cells and further cultured as previously described[1]. Early passage cultured cells were lysed and used for western blotting.

### Cell Culture, Transduction and ROS Measurement

Human aortic endothelial cells (HAECs) and human aortic smooth muscle cells (HASMCs) were purchased from Cascade Biologics and grown in endothelial cell basal medium-2 (Clonetics) or smooth muscle cell basal medium (Clonetics). COS-7 cells from ATCC were cultured in in Dulbecco’ s modified Eagle’s medium (DMEM) containing penicillin (100 U/ml), streptomycin (100 mg/ml), and 10% (v/v) fetal bovine serum. Mouse aortic endothelial and smooth muscle cells were isolated and cultured as previously described[1]. Replication-deficient adenoviruses encoding the control virus GFP or antisense-Bmal1 were generated and used to infect and deliver the gene construct in human aortic endothelial cells (HAEC) and human aortic smooth muscle cells (HASMC). Cells were incubated at 37°C, with 400 µmol/L L-012 (Wako) or 30 µmol/L Amplex Red. Luminescence (L012) and fluorescence (Amplex Red) was quantified over time using Lumistar Galaxy (BMG) luminometer.

### Statistical Analysis

One-way ANOVA with Tukey post-tests or two-way ANOVA with Bonferroni post-tests were used to determine significance in the experiments, analyzed through Graph pad Prism 5 (GraphPad Software Inc., La Jolla, CA) and cosinor analysis performed as described[19].

## Results

### Increased Nox4 Expression in Bmal1-KO mice

**Figure 1:**
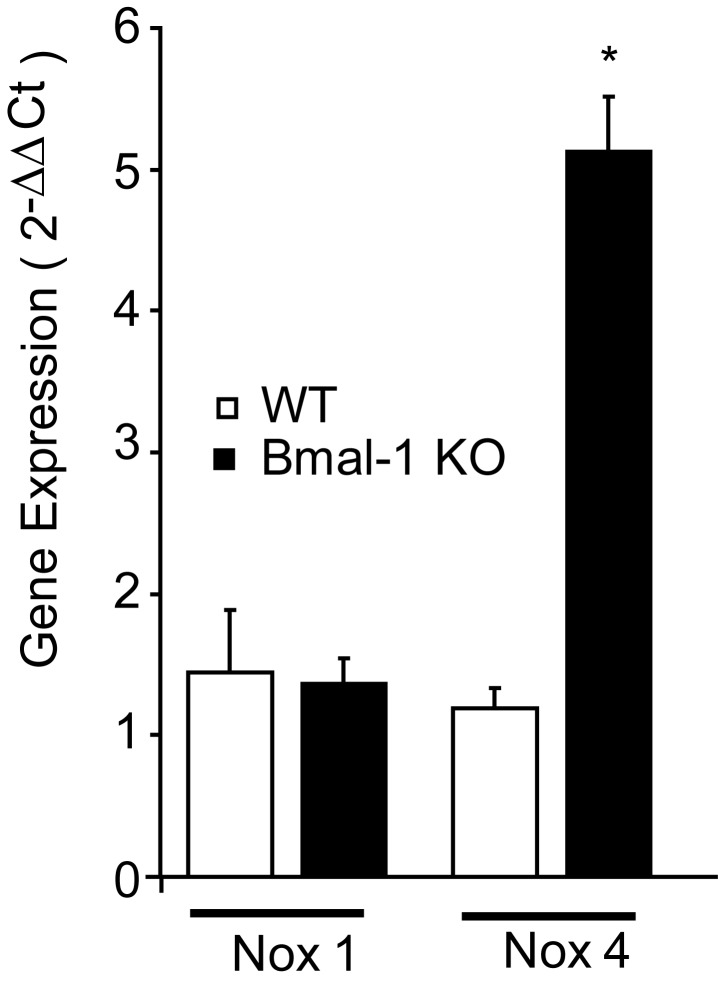
Increased Nox4 gene expression in aorta of Bmal1-KO mice. Aortae from WT and Bmal1-KO mice were isolated between ZT2 and ZT4, cryopreserved and total RNA isolated. Relative gene expression was assessed by qRT-PCR for Nox4 (forward primer, TGTTGCATGTTTCAGGTGGT; reverse, AAAACCCTCGAGGCAAAGAT) and Nox1(forward primer, CATGGCCTGGGTGGGATTGT; reverse, TGGGAGCGATAAAAGCGAAGGA) in mouse aorta and normalized to 18S. Bmal1-KO mice exhibited a significant increase in Nox4 gene expression (*P<0.05, n=6).

**Figure 2:**
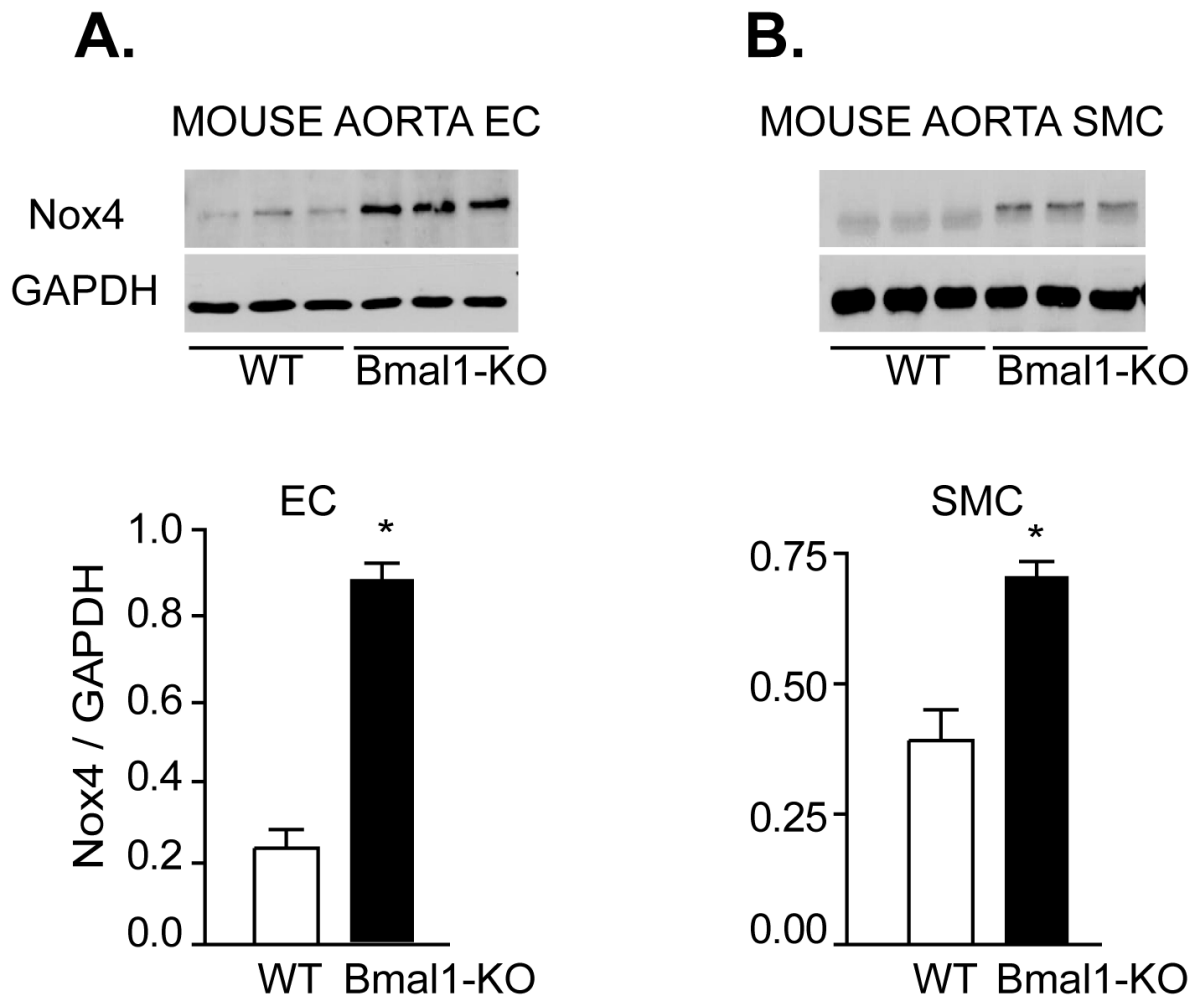
Increased Nox4 protein expression in cultured aortic endothelial and smooth muscle cells of Bmal1-KO mice. Vascular smooth muscle and endothelial cells were isolated and cultured from aortae of WT and Bmal1-KO mice (passage 2-3). Nox4 expression levels were determined by immunoblotting and revealed a significant increase in Nox4 in vascular endothelial cells (**A**) and smooth muscle cells (**B**) from Bmal1-KO animals relative to wild-type mice. Changes were quantified by densitometry (^*^p<0.05 versus WT, n=3).

To determine the interaction between circadian disruption and Nox4, we assessed Nox gene expression by quantitative real time -PCR in vascular tissue (aorta) of mice deficient for the Bmal1 gene. Expression levels for Nox4 were significantly elevated (3.5 fold increase) in aorta of Bmal1-KO mouse aorta versus wild type (WT) mice, while Nox1 expression did not vary significantly (Figure **1**). To dissect if there was differential expression of Nox4 controlled by Bmal1 in vascular cells, mouse aortic endothelial cells were isolated from Bmal1-KO mice. Nox4 expression was increased in both aortic endothelial cells (Figure **2A**) and smooth muscle cells (Figure **2B**). 

### Nox4 Promoter is Transactivated by the Circadian Clock

**Figure 3:**
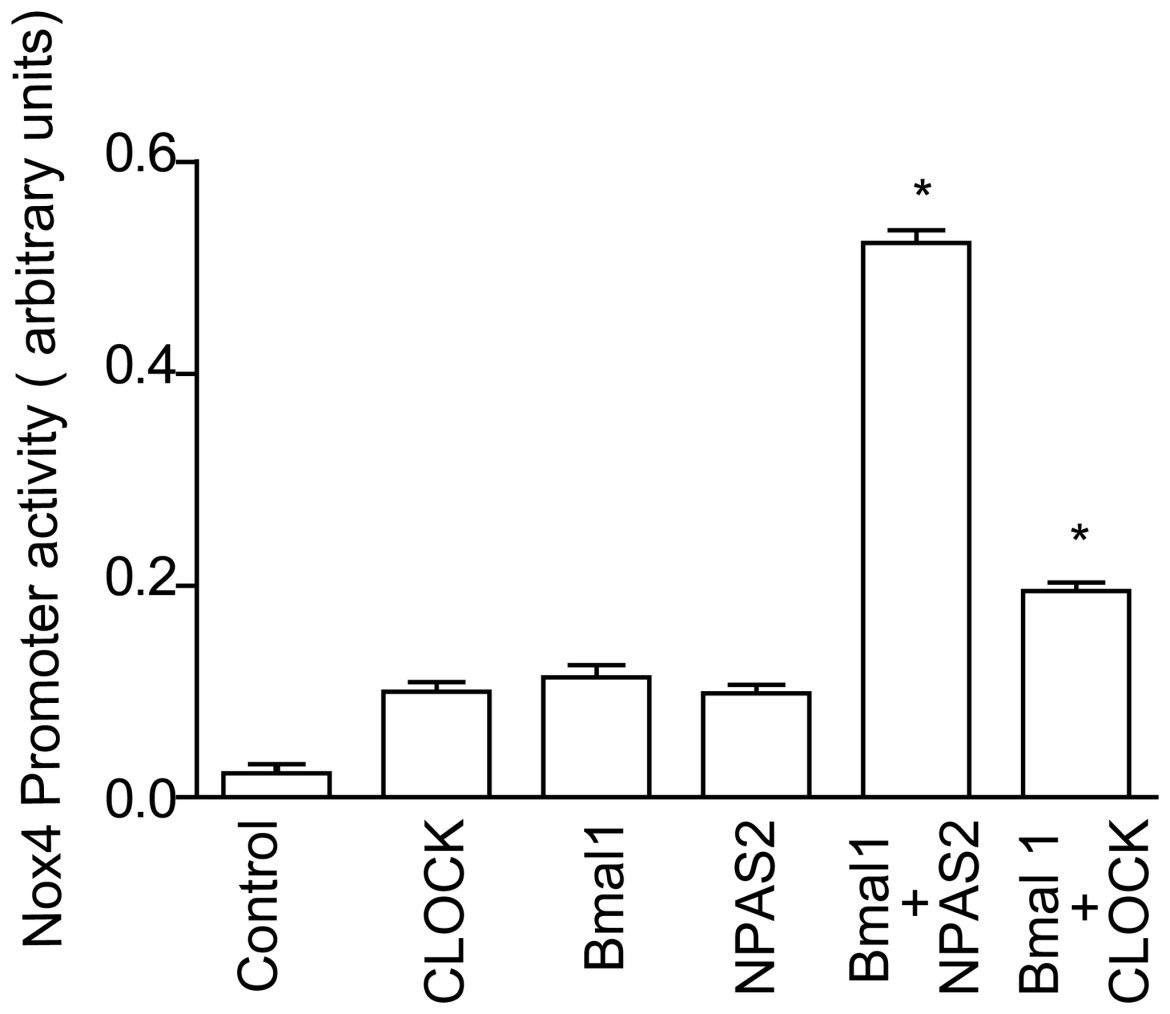
Nox4 promoter is regulated by the circadian clock. Human Nox4 promoter transactivation was assessed by a dual luciferase assay in transfected COS cells expressing the Nox4 promoter Gaussian luciferase in the presence and absence CLOCK, Bmal1, NPAS2, Bmal1+NPAS2 andBmal1+Clock. Cotransfection with Bmal1 and NPAS2 or Bmal1 and Clock significantly induced Nox4 promoter activity (*p<0.05 versus control, n=5).

To determine if the circadian clock might directly control the Nox4, we investigated promoter activity response of Nox4 to the circadian clock. It is well known that Bmal1 drives transcription of target genes by heterodimerizing with one of its basic helix-loop-helix protein partners, Npas2 or Clock. Thus, we conducted co-transfection studies using the human Nox4 promoter (subcloned in luciferase reporter vector) in COS cells with Bmal1 and either Clock or Npas2. Co-transfection of Bmal1 and Npas2 robustly activated the Nox4 promoter. Transfection of Bmal1 plus Clock also induced Nox4 promoter activity, albeit to a lesser extent (Figure **3**). As expected, Bmal1or Clock alone did not induce activation of the Nox4 promoter. These data demonstrate that Bmal1 plays an important role in the transcriptional regulation of Nox4.

### Elevated Hydrogen Peroxide caused by Bmal1 Suppression

**Figure 4:**
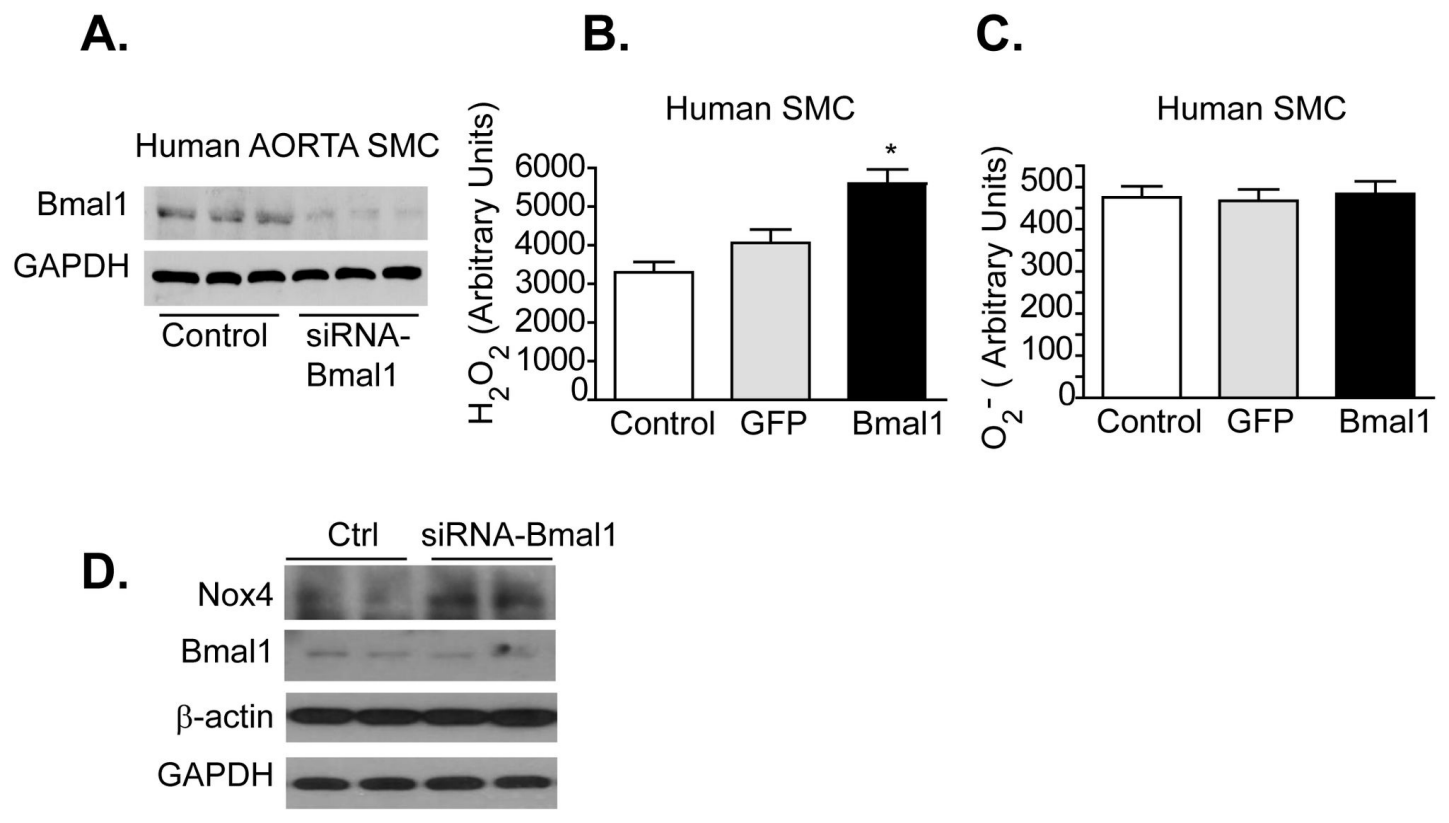
Bmal1 knockdown increases hydrogen peroxide in human aortic smooth muscle cells. (**A**) Western blot showing reduction in Bmal1 expression in human aortic smooth muscle cells incubated with antisense-Bmal1 adenovirus for 24 hours. (**B**) Knockdown of Bmal1 triggered an increase in H_2_O_2_ but (**C**) no detectable change in O_2_^. -^ levels in human aortic smooth muscle cells (HASMC). (n=8, *p<0.05) (**D**) Relative expression of Bmal1 and Nox4 versus B-actin and GAPDH in human aortic smooth muscle cells transfected with siRNA-Bmal1or control siRNA (30nM, n=3-5).

**Figure 5:**
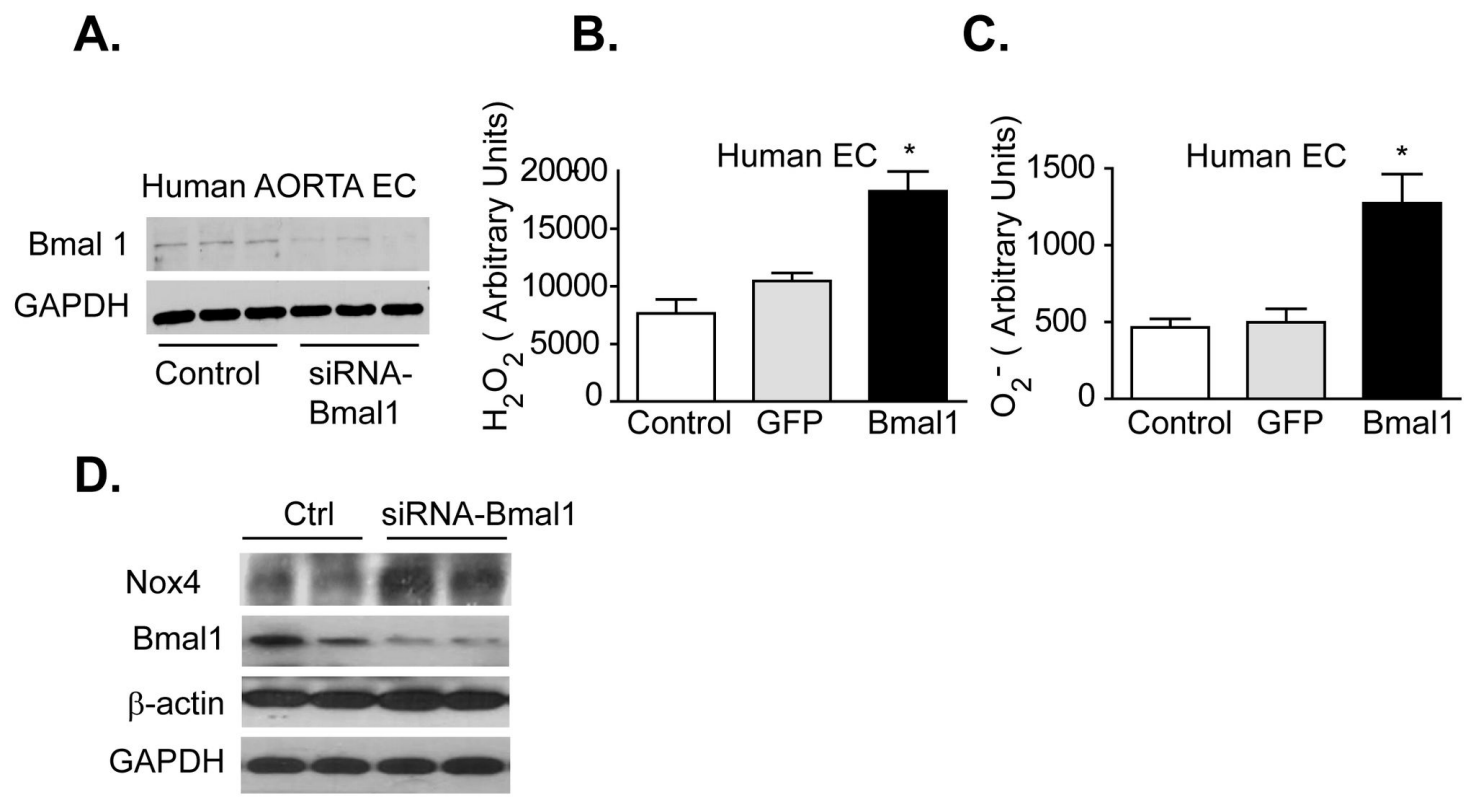
Bmal1 knockdown increases hydrogen peroxide and superoxide in human aortic endothelial cells. (**A**) Western blot showing reduction in Bmal1 expression in human aortic endothelial cells incubated with antisense-Bmal1 adenovirus. (**B**) Knockdown of Bmal1 resulted in increased H2O2 and superoxide (**C**) Relative expression of Bmal1 and Nox4 versus B-actin and GAPDH in human aortic endothelial cells transfected with siRNA-Bmal1or control siRNA (30nM, n=3-5). (**D**) Relative expression of Bmal1 and Nox4 versus β-actin and GAPDH in human aortic endothelial cells transfected with siRNA-Bmal1or control siRNA. Knockdown of Bmal1 increased Nox4 protein (n=8, *p<0.05 by one way ANOVA).

We next employed an antisense-Bmal1 knockdown strategy (as-Bmal1) in human aortic smooth muscle cells (HVSMC) and human aortic endothelial (HAEC) to investigate the impact of tissue specific circadian disruption on H_2_O_2_ and superoxide production. Suppression of Bmal1 (Figure **4A**) in HVSMCs resulted in increased hydrogen peroxide production (Figure **4B**), but had no effect on superoxide production (Figure **4C**). Knockdown of Bmal1 did however increase Nox4 protein expression in the smooth muscle cells (Figure **4D**). However, in HAECs, knockdown of Bmal1 (Figure **5A**) caused an increase in both hydrogen peroxide (Figure **5B**) and superoxide (Figure **5C**), while also increasing Nox4 expression (Figure **5D**). Indeed, recent work suggests that that the increase of endothelial superoxide caused by Bmal1 dysfunction may be in part due to uncoupling of eNOS [15].

### Circadian Oscillations in Nox4

**Figure 6:**
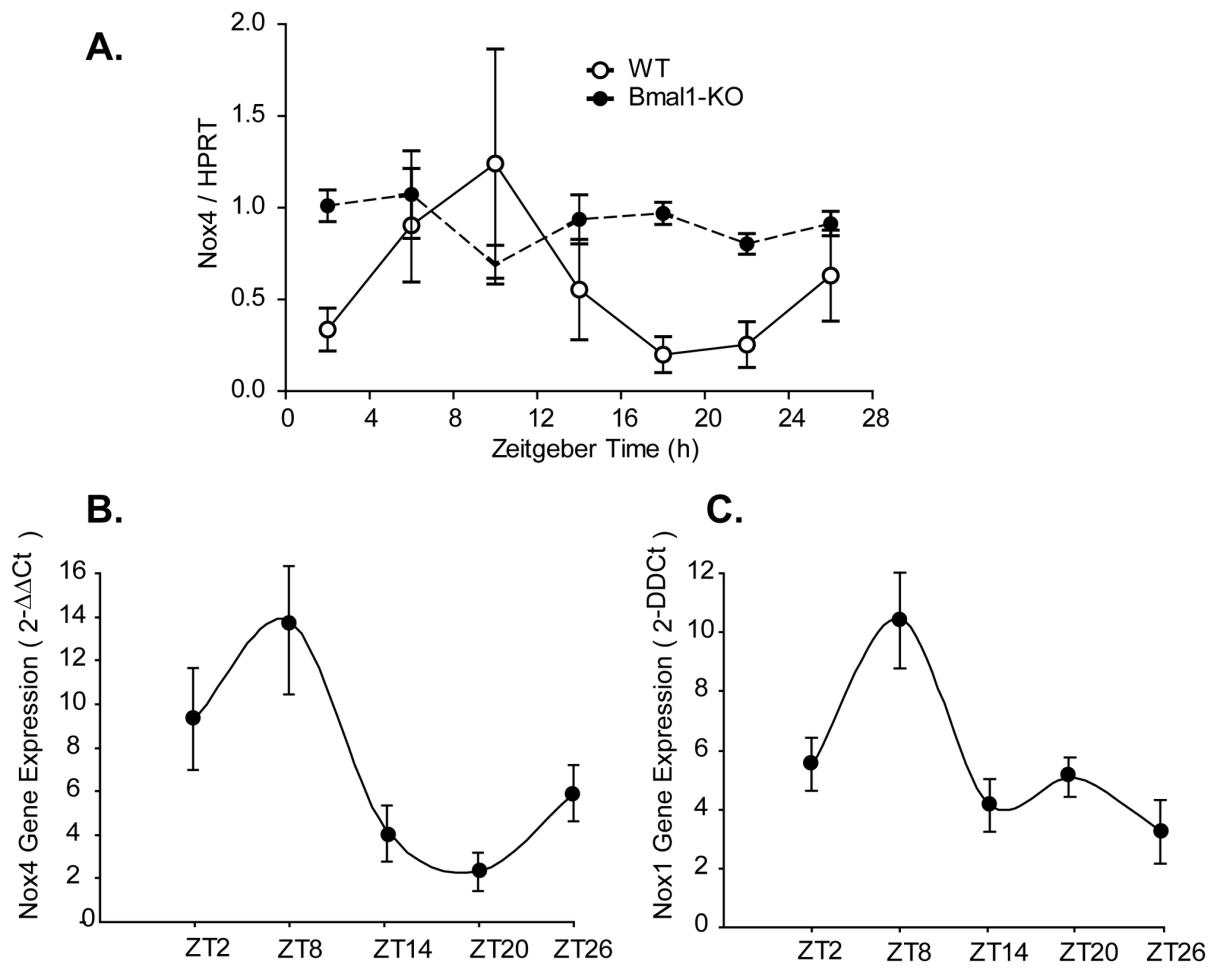
Circadian oscillation in Nox4 and Nox1 gene expression in cardiac and endothelial cells. (**A**)WT and Bmal1-KO hearts were isolated at 4 hour intervals, cryopreserved and RNA isolated. WT mice exhibited a significant rhythm in Nox4 expression as demonstrated by cosinor analysis (p=0.047), that was absent in Bmal1-KO mice (p=0.68). Circadian clocks were synchronized in human aortic endothelial cells by horse serum shock (20%), and cell lysates were harvested at 6-hour intervals for 24 hours. Expression levels of Nox4 (**B**) and Nox1 mRNA (**C**) at each time point were quantified by qRT-PCR. Nox4 and Nox1 exhibited a rhythmic expression pattern over 24 hours. (n=4-6).

Clock controlled genes are distinct in their expression pattern, having an oscillatory circadian profile with peaks and troughs within a 24 hour time period. To investigate whether Nox4 gene expression oscillates, we assessed tissue and cell expression over a time course. Indeed, Nox4 exhibited a circadian expression pattern in WT murine hearts, a rhythm that was absent in Bmal1-KO mice (Figure **6A**). In addition, assessment of overall expression levels revealed a significant increase in Nox4 expression in Bmal1-KO mice relative to the WT mice. We also examined oscillations in cultured cells. HAECs were subjected to serum shock, an established paradigm of circadian synchronization in cultured cells [20]. After serum shock, HAECs exhibited an oscillatory pattern in Nox4 (Figure **6B**) and Nox1 gene expression (Figure **6C**). These data suggest that Nox4 may exhibit an oscillatory circadian rhythm expression pattern, and that the circadian clock may play an important role in the control of Noxes resultant oxidant stress.

## Discussion

The circadian clock, a network of genes and proteins (including Bmal1, Clock, Period, and Cryptochrome genes) that controls 24-hour rhythms, is emerging as an important influence in the control of the cardiovascular system[1,2,21]. Acute vascular responses such as endothelial function[2,22], thrombogenesis[4], and blood pressure[23-25] have been demonstrated to be under control of the genetic components of the circadian clock. Also, chronic adaptation of the aging cardiovascular system is under clock control, including vascular remodeling [1,2,5] and angiogenesis[26]. Thus, the circadian clock may have an intricate entanglement into the etiology of cardiovascular disease. Indeed, there are human examples of circadian clock polymorphisms [27] that recapitulate the cardiovascular consequences of circadian clock dysfunction induced in mice by gene-targeted disruption of the circadian clock. In addition, epigenetic mechanisms also control circadian clock function[28], which may in part be a consequence of age-dependent worsening of vascular and circadian clock function. Our previous studies have demonstrated that the aging of blood vessels manifested by stiffening of the vasculature is accelerated in mice with circadian clock dysfunction[1], while other studies demonstrated that indeed aging in vascular cells and blood vessels impaired circadian clock expression[26,29]. Increasing oxidant stress which plays a key role in the process of aging [30] and deteriorating cardiovascular function[31] is also emerging as a significant mechanism controlled by the circadian clock to confer cardiovascular disease.

Recently we demonstrated that Bmal1-KO mice exhibit increased oxidant stress, and in particular superoxide, as a result of uncoupling of endothelial nitric oxide synthase[15]. This provoked us to determine if other oxidant stress signals might be affected or even controlled by the circadian clock. Indeed, in the current study, we have found that oxidant stress, in the form of hydrogen peroxide, is also increased by circadian clock dysfunction. Knockdown of Bmal1 in both cultured endothelial and smooth muscle cells increased hydrogen peroxide production, while knockdown of Bmal1 caused increased superoxide only in endothelial cells. These data suggest that Bmal1 influences the production of hydrogen peroxide in both endothelial and smooth muscle cells, while the effect of Bmal1 to control superoxide is endothelial-specific. Indeed, Nox4 is highly expressed in vascular cells and hydrogen peroxide release has been demonstrated in both endothelial [32,33] and smooth muscle cells[34-37]. Nox4 is unique among the Nox family in its ability to produce primarily hydrogen peroxide as a reactive oxygen species through mechanisms that are not entirely clear but are thought to involve modifications of the E-loop [38,39]. A further distinction is that Nox4 is regarded as an inducible (iNox) isoform that is constitutively active and regulated predominantly through changes in gene expression[40]. Our studies have found an unexpected Nox influence of the circadian clock through Bmal1 that impacts changes in Nox4 expression. In synchronized endothelial cells, Nox4 expression exhibited a 24-h rhythm which is consistent with the ~ 8% of genes under circadian control. Aortic tissue of Bmal1-KO mice exhibited increased Nox4 expression that was evident in both cultured endothelial and smooth muscle cells of Bmal1-KO mice. Interestingly, the human Nox4 promoter was strongly activated by co-transfection with Bmal1-Npas2 and more modestly by Bmal1-Clock. This is consistent with the activation mechanisms involving the circadian clock where by heterodimeric interactions between the basic-helix loop helix transcription factors which include Bmal1, Clock, and Npas2, are required for transactivation of the repressive limbs of the circadian clock, including the Per and Cryptochome (Cry) genes. While Bmal1/Npas2 cotransfection induced Nox4 promoter activity, this was in contrast to what was observed in Bmal1-KO mice which exhibited an increase in Nox4 expression. Such counterbalancing effects have also been observed with other circadian clock outputs/targets due to complex regulation involving the various circadian clock loops. For example, PAI-1 is regulated by three discrete circadian clock components. PAI-1 expression is driven by Bmal1 transactivation[41], inhibited by Rev-erbα[42] which is a repressor of Bmal1, and inhibited by the circadian Period-2[43]. In Bmal1-KO, PAI-1 expression is increased, with this complex array of regulatory signals. Thus, Nox4 may undergo additional complex regulation involving other clock components. More evidence to support the impact of the circadian clock on Nox4 expression is that many vascular signals that control the circadian clock also regulate Nox4. Nox4 is increased by TGF-β[44,45] , and suppressed by PPARγ agonists[46], 15-deoxy-Delta12,14-prostaglandin J2 (15d-PGJ2)[33], and Angiotensin II (AngII)[47]. Indeed, PPARγ[48], PGJ2[49], and Ang II[50] exhibit profound effects on the oscillation of the circadian clock [21]. Moreover, Nox4 was shown to be increased during smooth muscle differentiation after vascular injury [51]. Taken in conjunction with our findings that Nox4 is increased in Bmal1-KO mice, these results suggest that Nox4 may also be involved in vascular remodeling in conditions of circadian dysfunction [2]. While Nox4 mediated hydrogen peroxide production is known to both protect[52,53] and injure[54,55] the vasculature, an intriguing possibility is that the temporal oscillations in Nox4 may condition the ability of hydrogen peroxide to engage with effectors to confer such disparate outcomes. Indeed, though our data demonstrate a loss of circadian rhythmicity in Bmal1-KO mice, which over exhibit higher levels of Nox4 near the entire circadian cycle, WT mice exhibit a significant oscillation, where by Nox4 at discrete times of day is increased or decreased, which may differentially impact functional outputs of Nox4 signaling. 

While we have also previously shown the intrinsic importance of vascular Bmal1 and Period genes, our data also supports a direct role of the clock in control of Nox4 promoter. While it may be paradoxical that the promoter was transactivated by the circadian clock and that Bmal1-KO mice exhibited increased Nox4 expression, this may reflect several possibilities that will necessitate further investigation. Firstly, the difference in response to differences related to the human versus mouse Nox4 promoter mouse, though both contain a consensus E-box. It is also possible that in vivo, compensatory mechanisms or additional transcription factors may be recruited in conditions of Bmal1 disruption that may modify the response. 

In summary, we demonstrate elevated hydrogen peroxide in arteries of Bmal1-KO and in human endothelial and smooth muscle cells where Bmal1 expression is genetically silenced. We also demonstrate that Nox4 oscillates in serum shocked human endothelial cells, while Bmal1 and Clock transactivate the Nox4 promoter, suggesting that Nox4 may be a circadian output, directly controlled by the circadian clock. These data may provide new insight into the regulation of Nox4 expression and its ability to produce hydrogen peroxide and impact vascular function. 

## References

[1] Anea CB, Ali MI, Osmond JM, Sullivan JC, Stepp DW et al. (2010) Matrix metalloproteinase 2 and 9 dysfunction underlie vascular stiffness in circadian clock mutant mice. Arterioscler Thromb Vasc Biol 30: 2535-2543. doi:10.1161/ATVBAHA.110.214379. PubMed: 20829506. 20829506 PMC2988111

[2] Anea CB, Zhang M, Stepp DW, Simkins GB, Reed G et al. (2009) Vascular disease in mice with a dysfunctional circadian clock. Circulation 119: 1510-1517. doi:10.1161/CIRCULATIONAHA.108.827477. PubMed: 19273720. 19273720 PMC2761686

[3] Rudic RD, Curtis AM, Cheng Y, FitzGerald G (2005) Peripheral clocks and the regulation of cardiovascular and metabolic function. Methods Enzymol 393: 524-539. doi:10.1016/S0076-6879(05)93027-9. PubMed: 15817310. 15817310

[4] Westgate EJ, Cheng Y, Reilly DF, Price TS, Walisser JA et al. (2008) Genetic components of the circadian clock regulate thrombogenesis in vivo. Circulation 117: 2087-2095. doi:10.1161/CIRCULATIONAHA.107.739227. PubMed: 18413500. 18413500

[5] Cheng B, Anea CB, Yao L, Chen F, Patel V et al. (2011) Tissue-intrinsic dysfunction of circadian clock confers transplant arteriosclerosis. Proc Natl Acad Sci U S A 108: 17147-17152. doi:10.1073/pnas.1112998108. PubMed: 21969583. 21969583 PMC3193243

[6] Chatham JC, Young ME (2013) Regulation of myocardial metabolism by the cardiomyocyte circadian clock. J Mol Cell Cardiol 55: 139-146. doi:10.1016/j.yjmcc.2012.06.016. PubMed: 22766272. 22766272 PMC4107417

[7] Durgan DJ, Chess DJ, Tsai JY, Khairallah RJ, Pulinilkunnil T et al. (2010) The Cardiomyocyte Circadian Clock Modulates Responsiveness of the Heart to Hypertrophic Stimuli. Circulation 122.

[8] Durgan DJ, Pulinilkunnil T, Villegas-Montoya C, Garvey ME, Frangogiannis NG et al. (2010) Short communication: ischemia/reperfusion tolerance is time-of-day-dependent: mediation by the cardiomyocyte circadian clock. Circ Res 106: 546-U563. doi:10.1161/CIRCRESAHA.109.209346. PubMed: 20007913. 20007913 PMC3021132

[9] Richards J, Gumz ML (2013) Mechanism of the circadian clock in physiology. Am J Physiol Regul Integr Comp Physiol 304: R1053-R1064. doi:10.1152/ajpregu.00066.2013. PubMed: 23576606. 23576606 PMC4073891

[10] Schroder EA, Lefta M, Zhang X, Bartos DC, Feng HZ et al. (2013) The cardiomyocyte molecular clock, regulation of Scn5a, and arrhythmia susceptibility. Am J Physiol Cell Physiol 304: C954-C965. doi:10.1152/ajpcell.00383.2012. PubMed: 23364267. 23364267 PMC3651636

[11] Morris CJ, Yang JN, Scheer FA (2012) The impact of the circadian timing system on cardiovascular and metabolic function. Prog Brain Res 199: 337-358. doi:10.1016/B978-0-444-59427-3.00019-8. PubMed: 22877674. 22877674 PMC3704149

[12] Lefta M, Campbell KS, Feng HZ, Jin JP, Esser KA (2012) Development of dilated cardiomyopathy in Bmal1-deficient mice. Am J Physiol Heart Circ Physiol 303: H475-H485. doi:10.1152/ajpheart.00238.2012. PubMed: 22707558. 22707558 PMC3423146

[13] Su W, Xie Z, Guo Z, Duncan MJ, Lutshumba J et al. (2012) Altered clock gene expression and vascular smooth muscle diurnal contractile variations in type 2 diabetic db/db mice. Am J Physiol Heart Circ Physiol 302: H621-H633. doi:10.1152/ajpheart.00825.2011. PubMed: 22140039. 22140039 PMC3353796

[14] Grote K, Flach I, Luchtefeld M, Akin E, Holland SM et al. (2003) Mechanical stretch enhances mRNA expression and proenzyme release of matrix metalloproteinase-2 (MMP-2) via NAD(P)H oxidase-derived reactive oxygen species. Circ Res 92: e80-e86. doi:10.1161/01.RES.0000077044.60138.7C. PubMed: 12750313. 12750313

[15] Anea CB, Cheng B, Sharma S, Kumar S, Caldwell RW et al. (2012) Increased superoxide and endothelial NO synthase uncoupling in blood vessels of Bmal1-knockout mice. Circ Res 111: 1157-1165. doi:10.1161/CIRCRESAHA.111.261750. PubMed: 22912383. 22912383 PMC3740771

[16] Bedard K, Krause KH (2007) The NOX family of ROS-generating NADPH oxidases: Physiology and pathophysiology. Physiol Rev 87: 245-313. doi:10.1152/physrev.00044.2005. PubMed: 17237347. 17237347

[17] Martyn KD, Frederick LM, von Loehneysen K, Dinauer MC, Knaus UG (2006) Functional analysis of Nox4 reveals unique characteristics compared to other NADPH oxidases. Cell Signal 18: 69-82. doi:10.1016/j.cellsig.2005.03.023. PubMed: 15927447. 15927447

[18] Lu X, Murphy TC, Nanes MS, Hart CM (2010) PPAR{gamma} regulates hypoxia-induced Nox4 expression in human pulmonary artery smooth muscle cells through NF-{kappa}B. Am J Physiol Lung Cell Mol Physiol 299: L559-L566. doi:10.1152/ajplung.00090.2010. PubMed: 20622120. 20622120 PMC2957423

[19] Refinetti R, Lissen GC, Halberg F (2007) Procedures for numerical analysis of circadian rhythms. Biol Rhythm Res 38: 275-325. doi:10.1080/09291010600903692. PubMed: 23710111. 23710111 PMC3663600

[20] Balsalobre A, Damiola F, Schibler U (1998) A serum shock induces circadian gene expression in mammalian tissue culture cells. Cell 93: 929-937. doi:10.1016/S0092-8674(00)81199-X. PubMed: 9635423. 9635423

[21] Rudic RD (2009) Time is of the essence: vascular implications of the circadian clock. Circulation 120: 1714-1721. doi:10.1161/CIRCULATIONAHA.109.853002. PubMed: 19858424. 19858424 PMC2834195

[22] Viswambharan H, Carvas JM, Antic V, Marecic A, Jud C et al. (2007) Mutation of the circadian clock gene Per2 alters vascular endothelial function. Circulation 115: 2188-2195. doi:10.1161/CIRCULATIONAHA.106.653303. PubMed: 17404161. 17404161

[23] Curtis AM, Cheng Y, Kapoor S, Reilly D, Price TS et al. (2007) Circadian variation of blood pressure and the vascular response to asynchronous stress. Proc Natl Acad Sci U S A 104: 3450-3455. doi:10.1073/pnas.0611680104. PubMed: 17360665. 17360665 PMC1802007

[24] Gumz ML (2009) The circadian clock protein Period 1 regulates expression of the renal epithelial sodium channel in mice. J Clin Invest 119: 0-0. PubMed: 19587447. 10.1172/JCI36908 PMC2719945 19587447

[25] Masuki S, Todo T, Nakano Y, Okamura H, Nose H (2005) Reduced alpha-adrenoceptor responsiveness and enhanced baroreflex sensitivity in Cry-deficient mice lacking a biological clock. J Physiol 566: 213-224. doi:10.1113/jphysiol.2005.086728. PubMed: 15860530. 15860530 PMC1464725

[26] Wang CY, Wen MS, Wang HW, Hsieh IC, Li Y et al. (2008) Increased vascular senescence and impaired endothelial progenitor cell function mediated by mutation of circadian gene Per2. Circulation 118: 2166-2173. doi:10.1161/CIRCULATIONAHA.108.790469. PubMed: 18981300. 18981300 PMC2656770

[27] Woon PY, Kaisaki PJ, Bragança J, Bihoreau MT, Levy JC et al. (2007) Aryl hydrocarbon receptor nuclear translocator-like (BMAL1) is associated with susceptibility to hypertension and type 2 diabetes. Proc Natl Acad Sci U S A 104: 14412-14417. doi:10.1073/pnas.0703247104. PubMed: 17728404. 17728404 PMC1958818

[28] Bellet MM, Sassone-Corsi P (2010) Mammalian circadian clock and metabolism – the epigenetic link. J Cell Sci 123: 3837-3848. doi:10.1242/jcs.051649. PubMed: 21048160. 21048160 PMC2972271

[29] Matthews C, Gorenne I, Scott S, Figg N, Kirkpatrick P et al. (2006) Vascular smooth muscle cells undergo telomere-based senescence in human atherosclerosis: effects of telomerase and oxidative stress. Circ Res 99: 156-164. doi:10.1161/01.RES.0000233315.38086.bc. PubMed: 16794190. 16794190

[30] Finkel T, Holbrook NJ (2000) Oxidants, oxidative stress and the biology of ageing. Nature 408: 239-247. doi:10.1038/35041687. PubMed: 11089981. 11089981

[31] Cai H, Harrison DG (2000) Endothelial dysfunction in cardiovascular diseases: the role of oxidant stress. Circ Res 87: 840-844. doi:10.1161/01.RES.87.10.840. PubMed: 11073878. 11073878

[32] Carter WO, Narayanan PK, Robinson JP (1994) Intracellular hydrogen peroxide and superoxide anion detection in endothelial cells. J Leukoc Biol 55: 253-258. PubMed: 8301222. 8301222 10.1002/jlb.55.2.253

[33] Hwang J, Kleinhenz DJ, Lassègue B, Griendling KK, Dikalov S et al. (2005) Peroxisome proliferator-activated receptor-gamma ligands regulate endothelial membrane superoxide production. Am J Physiol Cell Physiol 288: C899-C905. doi:10.1152/ajprenal.00370.2004. PubMed: 15590897. 15590897

[34] Cheng G, Cao Z, Xu X, Van Meir EG, Lambeth JD (2001) Homologs of gp91phox: cloning and tissue expression of Nox3, Nox4, and Nox5. Gene 269: 131-140. doi:10.1016/S0378-1119(01)00449-8. PubMed: 11376945. 11376945

[35] Griendling KK, Sorescu D, Ushio-Fukai M (2000) NAD(P)H oxidase: role in cardiovascular biology and disease. Circ Res 86: 494-501. doi:10.1161/01.RES.86.5.494. PubMed: 10720409. 10720409

[36] Sundaresan M, Yu ZX, Ferrans VJ, Irani K, Finkel T (1995) Requirement for generation of H2O2 for platelet-derived growth factor signal transduction. Science 270: 296-299. doi:10.1126/science.270.5234.296. PubMed: 7569979. 7569979

[37] Touyz RM, Chen X, Tabet F, Yao G, He G et al. (2002) Expression of a functionally active gp91phox-containing neutrophil-type NAD(P)H oxidase in smooth muscle cells from human resistance arteries: regulation by angiotensin II. Circ Res 90: 1205-1213. doi:10.1161/01.RES.0000020404.01971.2F. PubMed: 12065324. 12065324

[38] Drummond GR, Selemidis S, Griendling KK, Sobey CG. (2011) Combating oxidative stress in vascular disease: NADPH oxidases as therapeutic targets. Nat Rev Drug Discov 10: 453-471. PubMed: 21629295. 21629295 10.1038/nrd3403 PMC3361719

[39] Takac I, Schröder K, Zhang L, Lardy B, Anilkumar N et al. (2011) The E-loop is involved in hydrogen peroxide formation by the NADPH oxidase Nox4. J Biol Chem 286: 13304-13313. doi:10.1074/jbc.M110.192138. PubMed: 21343298. 21343298 PMC3075677

[40] Chen F, Haigh S, Barman S, Fulton DJ (2012) From form to function: the role of Nox4 in the cardiovascular system. Front. Physiol (Bethesda Md.) 3: 412. 10.3389/fphys.2012.00412 PMC3485577 23125837

[41] Schoenhard JA, Smith LH, Painter CA, Eren M, Johnson CH et al. (2003) Regulation of the PAI-1 promoter by circadian clock components: differential activation by BMAL1 and BMAL2. J Mol Cell Cardiol 35: 473-481. doi:10.1016/S0022-2828(03)00051-8. PubMed: 12738229. 12738229

[42] Wang J, Yin L, Lazar MA (2006) The Orphan Nuclear Receptor Rev-erbα Regulates Circadian Expression of Plasminogen Activator Inhibitor Type 1. Journal of Biological Chemistry 281: 33842-33848. 16968709 10.1074/jbc.M607873200

[43] Oishi K, Miyazaki K, Uchida D, Ohkura N, Wakabayashi M et al. (2009) PERIOD2 is a circadian negative regulator of PAI-1 gene expression in mice. J Mol Cell Cardiol 46: 545-552. doi:10.1016/j.yjmcc.2009.01.001. PubMed: 19168071. 19168071

[44] Cucoranu I, Clempus R, Dikalova A, Phelan PJ, Ariyan S et al. (2005) NAD(P)H oxidase 4 mediates transforming growth factor-beta1-induced differentiation of cardiac fibroblasts into myofibroblasts. Circ Res 97: 900-907. doi:10.1161/01.RES.0000187457.24338.3D. PubMed: 16179589. 16179589

[45] Hecker L, Vittal R, Jones T, Jagirdar R, Luckhardt TR et al. (2009) NADPH oxidase-4 mediates myofibroblast activation and fibrogenic responses to lung injury. Nat Med 15: 1077-1081. doi:10.1038/nm.2005. PubMed: 19701206. 19701206 PMC2743335

[46] Nisbet RE, Bland JM, Kleinhenz DJ, Mitchell PO, Walp ER et al. (2010) Rosiglitazone attenuates chronic hypoxia-induced pulmonary hypertension in a mouse model. Am J Respir Cell Mol Biol 42: 482-490. doi:10.1165/rcmb.2008-0132OC. PubMed: 19520921. 19520921 PMC2848739

[47] Chabrashvili T, Kitiyakara C, Blau J, Karber A, Aslam S et al. (2003) Effects of ANG II type 1 and 2 receptors on oxidative stress, renal NADPH oxidase, and SOD expression. Am J Physiol Regul Integr Comp Physiol 285: R117-R124. PubMed: 12609817. 12609817 10.1152/ajpregu.00476.2002

[48] Wang N, Yang G, Jia Z, Zhang H, Aoyagi T et al. (2008) Vascular PPARgamma controls circadian variation in blood pressure and heart rate through Bmal1. Cell Metab 8: 482-491. doi:10.1016/j.cmet.2008.10.009. PubMed: 19041764. 19041764 PMC5484540

[49] Koinuma S, Yagita K, Fujioka A, Takashima N, Takumi T et al. (2009) The resetting of the circadian rhythm by Prostaglandin J2 is distinctly phase-dependent. FEBS Lett 583: 413-418. doi:10.1016/j.febslet.2008.12.035. PubMed: 19111547. 19111547

[50] Nonaka H, Emoto N, Ikeda K, Fukuya H, Rohman MS et al. (2001) Angiotensin II induces circadian gene expression of clock genes in cultured vascular smooth muscle cells. Circulation 104: 1746-1748. doi:10.1161/hc4001.098048. PubMed: 11591607. 11591607

[51] Lassègue B, Clempus RE (2003) Vascular NAD(P)H oxidases: specific features, expression, and regulation. Am J Physiol Regul Integr Comp Physiol 285: R277-R297. PubMed: 12855411. 12855411 10.1152/ajpregu.00758.2002

[52] Zhang M, Brewer AC, Schröder K, Santos CX, Grieve DJ et al. (2010) NADPH oxidase-4 mediates protection against chronic load-induced stress in mouse hearts by enhancing angiogenesis. Proc Natl Acad Sci U S A 107: 18121-18126. doi:10.1073/pnas.1009700107. PubMed: 20921387. 20921387 PMC2964252

[53] Schröder K, Zhang M, Benkhoff S, Mieth A, Pliquett R et al. (2012) Nox4 is a protective reactive oxygen species generating vascular NADPH oxidase. Circ Res 110: 1217-1225. doi:10.1161/CIRCRESAHA.112.267054. PubMed: 22456182. 22456182

[54] Sundaresan M, Yu ZX, Ferrans VJ, Irani K, Finkel T (1995) Requirement for generation of H2O2 for platelet-derived growth factor signal transduction. Science 270: 296-299. doi:10.1126/science.270.5234.296. PubMed: 7569979. 7569979

[55] Zafari AM, Ushio-Fukai M, Akers M, Yin Q, Shah A et al. (1998) Role of NADH/NADPH oxidase-derived H2O2 in angiotensin II-induced vascular hypertrophy. Hypertension 32: 488-495. doi:10.1161/01.HYP.32.3.488. PubMed: 9740615. 9740615

